# Tree Resin Composition, Collection Behavior and Selective Filters Shape Chemical Profiles of Tropical Bees (Apidae: Meliponini)

**DOI:** 10.1371/journal.pone.0023445

**Published:** 2011-08-08

**Authors:** Sara D. Leonhardt, Thomas Schmitt, Nico Blüthgen

**Affiliations:** 1 Department of Animal Ecology and Tropical Biology, University of Würzburg, Würzburg, Germany; 2 Department of Evolutionary Biology and Animal Ecology, Faculty of Biology, University of Freiburg, Freiburg, Germany; Royal Holloway University of London, United Kingdom

## Abstract

The diversity of species is striking, but can be far exceeded by the chemical diversity of compounds collected, produced or used by them. Here, we relate the specificity of plant-consumer interactions to chemical diversity applying a comparative network analysis to both levels. Chemical diversity was explored for interactions between tropical stingless bees and plant resins, which bees collect for nest construction and to deter predators and microbes. Resins also function as an environmental source for terpenes that serve as appeasement allomones and protection against predators when accumulated on the bees' body surfaces. To unravel the origin of the bees' complex chemical profiles, we investigated resin collection and the processing of resin-derived terpenes. We therefore analyzed chemical networks of tree resins, foraging networks of resin collecting bees, and their acquired chemical networks. We revealed that 113 terpenes in nests of six bee species and 83 on their body surfaces comprised a subset of the 1,117 compounds found in resins from seven tree species. Sesquiterpenes were the most variable class of terpenes. Albeit widely present in tree resins, they were only found on the body surface of some species, but entirely lacking in others. Moreover, whereas the nest profile of *Tetragonula melanocephala* contained sesquiterpenes, its surface profile did not. Stingless bees showed a generalized collecting behavior among resin sources, and only a hitherto undescribed species-specific “filtering” of resin-derived terpenes can explain the variation in chemical profiles of nests and body surfaces from different species. The tight relationship between bees and tree resins of a large variety of species elucidates why the bees' surfaces contain a much higher chemodiversity than other hymenopterans.

## Introduction

Biodiversity is considered a crucial feature of ecosystems worldwide, by, for instance, providing a variety of organisms that maintain ecosystem functioning and services [1]. The higher the diversity of species in a habitat, the more interactions occur between them, resulting in complex interaction networks [2]–[4]. Here, we used a plant-insect interaction network to unravel the origin of a rather neglected kind of diversity: chemical diversity – describing the heterogeneity of chemical compounds produced or acquired and used by organisms. The reliance on such chemical compounds is particularly pronounced in plants and insects.

Plants produce secondary metabolites to defend themselves against herbivores [5] or to attract mutualists, such as parasitoids [6], [7] and pollinators [8]–[11]. The composition of secondary metabolites may vary across seasons [12], developmental states [12], [13], species [11], [14], individuals, different plant parts of the same individual [15], [16] or in response to herbivore attack [6], [7].

Insects use chemical compounds to recognize potential mates, relatives, nestmates or enemies, but also to mark suitable nesting sites or resources and to defend themselves against predators [17]–[19]. Qualitative and quantitative differences between chemical mixtures/bouquets usually indicate different species [20]–[23]. Within species, quantitative differences between compounds signify different colonies, ages, genders, castes and/or differences in the reproductive status of individuals [24]–[28].

The large number of functions and meanings mediated by chemical compounds is thus associated with a chemical heterogeneity that far exceeds the diversity of plants and insects themselves, because even conspecific individuals may have different chemical profiles due to quantitative variation.

Insects synthesize chemical compounds *de novo* in specialized glands (genetically determined compounds; [29], [30]–[32]) and/or acquire compounds from the environment – predominantly from plants. For instance, euglossine bees collect various volatiles from flowers or other plant parts [33], [34], and some specialized herbivores sequester defensive compounds from their host plant (e.g.; resin terpenoids in sawfly larvae: [35], alkaloids in butterflies: [36]). Chemical profiles of insects can therefore represent a mixture of both genetically determined and plant-derived compounds [20], [37], thereby increasing the diversity and heterogeneity of compounds available for communication and/or defense. The secondary metabolites of plants can thus be tracked along the food chain, in which the specificity of plant-insect interactions mediates the distribution of plant compounds among insects.

We here focus on the origin of plant-derived chemical compounds in tropical stingless bees (Meliponini). Stingless bees have eusocial colonies and are considered crucial pollinators in tropical forests [38], [39]. Besides pollen and nectar, they also collect large amounts of plant resins for nest construction and defense [39], [40]. Terpenes likely derived from these resins seem to be transferred to the bees' body surfaces (chemical profiles), where they are mixed with self-produced non-terpenoid compounds (non-polar aliphatic compounds, alcohols, aldehydes and esters) [20]. Notably, different bee species strongly differ in their terpene profiles with entire classes of terpenes being present in some and absent in other species [20]. Terpenes were also found on the bees' wings, rendering mere contamination by resin highly unlikely [20]. The terpenes on the bees' surfaces repel predators (ants, [41]) and reduce interspecific aggression [42].

We attempt to reveal how the bees' foraging behavior and the chemical diversity of tree resins affect the chemical diversity of their surface profiles. We thereby link behavior and chemistry by applying two-dimensional network analyses [43] to both species – interaction (foraging) networks and compound – species (chemical) networks. By observing bees at trees (sources of chemical compounds) and nest entrances, we investigated whether different stingless bee species collected resin from different tree species (specialized) or from the same tree species (generalized). If bees merely transferred resin-derived terpenes to their surfaces without altering their composition, we would expect that species-specificity of resin collection would directly predict the specificity of their chemical profiles. In addition to the behavioral observations, we therefore analyzed and compared the chemical profiles of tree resins, nest and bee profiles with regard to resin derived terpenes and non-terpenoid compounds, in order to track terpenes from tree resins to the bees' profiles. Moreover, to investigate whether the acquisition of resin-derived compounds increases the diversity of surface compounds in stingless bees beyond the diversity of compounds normally found on the body surfaces of (social) insects, we compared the chemical diversity of stingless bees with that of other hymenopterans and discuss the contributions of plant derived and genetically determined compounds.

## Methods

### Study sites and bees

Fieldwork was performed in Borneo (Malaysia), from March 2006 to November 2008. Observations and sample collection took part at the Danum Valley Conservation Area (DVC: Sabah, 4°55′N 117°40′E, 100 m asl), the Kabili Sepilok Reserve (KSR: Sabah, 5°54′N, 118°04′E, 20–120 m asl) and the Rainforest discovery centre (RDC). DVC represents one of the major remaining patches of Sabah's primary lowland dipterocarp rainforest (43 800 ha) [44]. KSR comprises 4294 ha of coastal dipterocarp and mangrove forest [45] and the RDC is a small (148.6 ha) education centre about 2 km west of KSR.

About fifteen stingless bee species (species and genus names as in [46]) have been reported for DVC [47]. In KSR and RDC, 15 to 20 species can be found according to collections of specimens held by the Forestry Research Centre in Sepilok and our own studies [48].

### Foraging networks: Observation of resin collection at trees and at nest entrances

To analyze the degree of specialization on resin sources in stingless bees, we observed bees collecting resin from wounds of 60 tree individuals in total (15 tree species belonging to five tree families, with 75% of the trees representing dipterocarps, Table 1) at the RDC, in August 2008. Observations comprised five natural and 55 artificially induced resin wounds. Natural wounds were relatively common at the RDC and comprised wounds caused by fresh branch breakage or insects (e.g., wood burrowing beetles), and wounds due to spontaneous bleeding [15]. Bees are frequently visiting resin wounds of some tree species but entirely neglect others, even when several artificial wounds were offered at the same site [48]. Hence, our artificially induced wounds should barely (if at all) influence natural foraging patterns of resin collecting bees. Artificial resin wounds were inflicted to trees by either hammering nails in the trunk or cutting the trees' bark with a machete. We noted the number of bee species collecting resin at a given resin wound following wound insertion (artificial wounds) or wound discovery (natural wounds) for an observation period of 2–5 minutes (Table 1). Each bee collecting resin during the observation period was counted only once.

**Table 1:** Bee and tree species represented in the foraging and chemical networks.

Name code	Species name	Family	N1	N2	N3
Trees					
*AB*	*Agathis borneensis*	Araucariaceae	8	1	-
*CO*	*Canarium odontophylum*	Bursearceae	2	-	-
*DA*	*Dipterocarpus applanatus*	Dipterocarpaceae	1	-	-
*DL*	*Dryobalanops lanceolata*	Dipterocarpaceae	7	1	-
*DS*	*Dacryodes* spec.	Bursearceae	1	1	-
*DSt*	*Dipterocarpus stellatus*	Dipterocarpaceae	1	-	-
*GS*	*Gluta sabatan*	Anacardiaceae	1	-	-
*HN*	*Hopea nervosa*	Dipterocarpaceae	1	3	-
*KS*	*Knema* spec.	Myristicacea	2	-	-
*PM*	*Parashorea melanonaan*	Dipterocarpaceae	2	3	-
*PT*	*Parashorea tomentella*	Dipterocarpaceae	11	1	-
*SM*	*Shorea macroptera*	Dipterocarpaceae	7	-	-
*SP*	*Shorea parvifolia*	Dipterocarpaceae	1	2	-
*SS*	*Shorea smithiana*	Dipterocarpaceae	4	2	-
*SX*	*Shorea xantophylla*	Dipterocarpaceae	13	-	-
Bees					
*GT*	*Geniotrigona thoracica*	Apidae	6	-	-
*HE*	*Heterotrigona erythrogaster*	Apidae	1	-	-
*HF*	*Homotrigona fimbriata*	Apidae	10	-	-
*LC*	*Lophotrigona canifrons*	Apidae	13	-	-
*LT*	*Lepidotrigona terminata*	Apidae	-	21	1
*OH*	*Odontotrigona haematoptera*	Apidae	2	-	-
*PH*	*Platytrigona hobbyi*	Apidae	1	-	-
*PP*	*Pariotrigona pendleburyi*	Apidae	-	8	1
*TA*	*Tetrigona apicalis*	Apidae	6	-	-
*TB*	*Tetrigona binghami*	Apidae	21	-	-
*TC*	*Tetragonilla collina*	Apidae	25	29	2
*TF*	*Tetragonula fuscobalteata*	Apidae	-	79	5
*TG1*	*Tetragonula geissleri/laeviceps* group 1	Apidae	11	-	-
*TG2*	*Tetragonula geissleri/laeviceps* group 2	Apidae	10	19	2
*TM*	*Tetragonula melanocephala*	Apidae	7	9	4
*TR*	*Tetragonilla rufibasalis*	Apidae	2	-	-

N1 gives the number of trees of a particular species (top part of table) or number of bees of a particular species (bottom part of table) observed in the foraging network, N2 gives the number of tree individuals of a particular species (top part of table) or number of bee specimens of a particular species (bottom part of table) analyzed for the chemical networks and N3 gives the number of nests from which material was analyzed for a given bee species.

We also attempted to estimate foraging specialization for resin at the bee colony level, i.e. the heterogeneity of resin sources brought into a colony by a large number of foragers. We assume that each resin forager uses only a single resin source, as resin sources are relatively large and usually contain sufficient resin to fully load both hindlegs. Resin collecting bees were monitored directly at nest entrances of two species (*Tetragonilla collina*, 4 nests; *Tetragonula melanocephala*, 4 nests) in 2007 and three species (*T. collina*, 6 nests; *T. melanocephala*, 3 nests; *Tetragonula geissleri/laeviceps* group, 2 nests) in 2008. We recorded the number of resin foragers carrying resin of a particular color as a method to estimate the variety and uniqueness of resin sources brought into a colony. We defined 25 different color patterns for resin (including white, yellow, red, black, brown and opaque resin with different varieties of these colors, e.g. light-brown and dark brown). Resin color was assessed by eye. Each nest was observed at different times of the day and between ten and 40 times in total to ensure that a large spectrum of daily resin foragers was recorded. We collected resin from 772 foragers of *T. collina* (6 nests), 142 of *T. melanocephala* (3 nests) and 35 of the *T. geissleri/laeviceps* group (2 nests) for color identification (in 2007 and 2008). Counting resin colors represents a rather conservative approach, because different tree species may have resin of the same color, while resin color is relatively constant across different individuals of the same tree species. According to our own observations, resin color only slightly changes over time. Also, bees tend to mainly collect fresh resin which has not hardened yet. Therefore, resin collected from the bees' corbiculae most likely represents fresh (or middle-aged), but unlikely old resin.

### Chemical networks: Collection of bee-, nest- and resin-samples and chemical analysis

**Figure 1:**
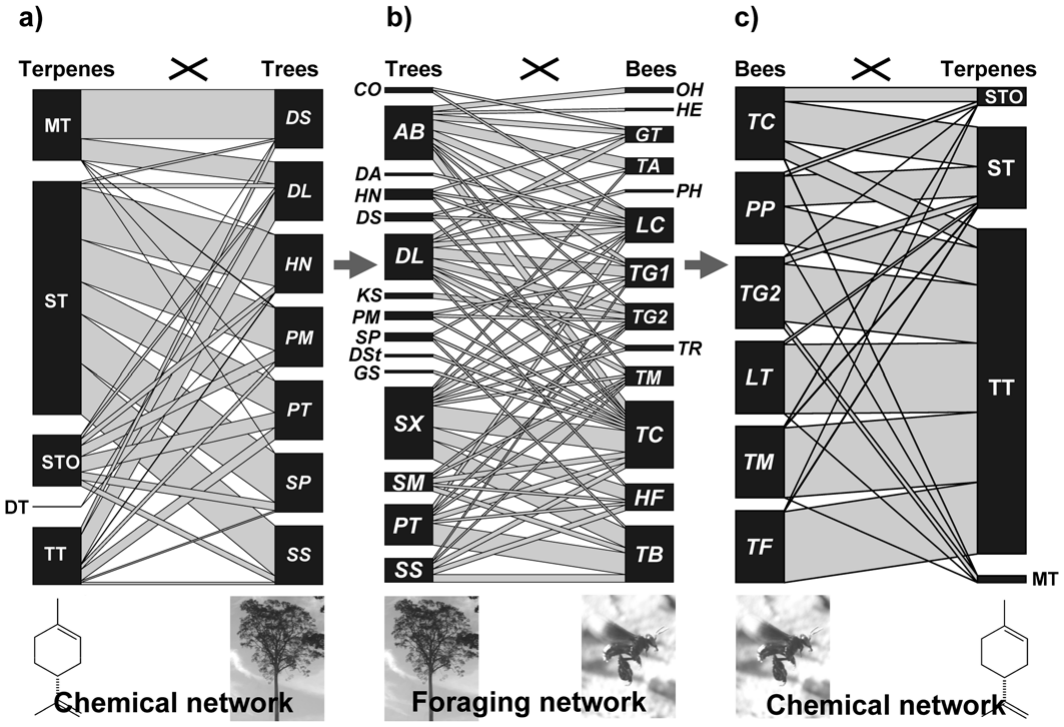
Chemical and foraging networks, representing (a) seven tree species and the terpenes of their resins (MT = monoterpenes, ST = sesquiterpenes without functional groups, STO = sesquiterpenes with functional groups, DT = diterpenes, TT = triterpenes), (b) 15 tree species and 13 bee species collecting resin at these trees, and (c) terpenes found on the body surface of six bee species. Note that resin samples could not be analyzed for all tree species visited by bees and that nests were only found for six bee species, limiting the number of bee species whose chemical profiles were analyzed. Names of bee and tree species are given in Table 1. Block sizes represent overall proportions of species or terpene groups (based on mean relative amount of compounds) within a given network. Note that the chemical compounds of each tree and bee species add up to 100%, hence their block sizes are equal.

The chemical profiles of 3–13 bees from 31 colonies (six species, Table 1) sampled in 2006 were analyzed as described in Leonhardt et al. [20]. We compared the terpene composition of the bees' surfaces with their nest material from a subset of 15 colonies (including all six species, Table 1) and with resin samples from a subset of 14 trees (seven species, Table 1, Fig. 1) all of them visited by bees for resin collection. Nest material was collected as described in Leonhardt et al. [49]: by breaking off small pieces from the bees' nest entrance tubes. Fresh resin samples were obtained directly from natural or artificially induced resin wounds studied in 2007 (see [48]).

If bees were able to modify the composition of resin-derived compounds, they could do so by e.g. adding specific enzymes or with the help of mutualistic microbes either directly during resin collection at trees or later inside their nests. We thus additionally collected resin from the hindlegs (corbiculae) of five *T. collina* foragers gathering resin from an *Agathis borneensis* tree (Araucariaceae). The resin from corbiculae was processed directly or after having been stored in a plastic bag for 0, 1, 3, 6 and 12 h to see whether its chemical composition changed with time. For comparison with resin not touched by the bee, five resin samples from the same tree were obtained manually and stored for 0, 1, 6, 12 and 20 h.

Approximately 500 mg nest material and 0.4 ml resin were transferred into 2 ml sample vials containing 1.5 ml pure hexane. We analyzed the solvable components of these materials using a Hewlett Packard HP 6890 Series gas chromatography (GC) System coupled to a Hewlett Packard HP 5973 Mass Selective Detector (Agilent Technologies, Böblingen, Germany). The components were characterized in the same way as described in Leonhardt et al. [20] for the components of the bees' chemical profiles: by comparing their mass spectra and retention times with mass spectra from three commercially available libraries (Wiley 275, NIST 98 and Adams EO library 2205), and by comparing them to synthetic standards (Sigma-Aldrich, Munich, Germany) if standards were available. For statistical analyses, we used only compounds that accounted for at least 0.05% of the total peak area in at least one sample. Overall, we analyzed 1117 resin-compounds, 247 nest-compounds and 194 bee-compounds. The following substance classes were determined: non-polar aliphatic compounds (alkanes, alkenes, alkadienes and methylated alkanes/alkenes), oxygenated aliphatic compounds (aldehydes and alcohols), esters, monoterpenes, (methylated) sesquiterpenes, oxygenated sesquiterpenes, diterpenes and putatively identified triterpenes. Across nests, bee profiles and resin samples, we regarded peaks with the same mass spectra and retention times as the same compound.

To test for potential changes in the chemical composition of resin samples from trees to bee legs, approximately 0.1 ml resin from corbiculae of *T. collina* foragers as well as from the bees' collecting tree (*A. borneensis*) was fractionated to obtain polar compounds. Polar compounds have been found in large amounts in tree resins but only in traces on the cuticle of bees (see also [20]), and are more likely to be targeted by enzymes potentially added by the bees. We used 6 ml SiOH polypropylene columns (CHROMABOND®, 500 mg, Macherey-Nagel, Düren, Germany) that were conditioned with pentane before adding about 40 µl of surface extract. Non-polar and polar fractions of extracts were eluted with 2 column equivalents of hexane and subsequently with 3 column equivalents of dichloromethane. Success of fractionation was controlled by GC-MS. We then compared the chemical composition of polar compounds in resin samples collected from bee corbiculae and resin samples collected directly from *A. borneensis* using an Adonis test, a multi-response permutation procedure for a randomization-based analysis of dissimilarities (library vegan in R, R Foundation for Statistical Computing, version 2009, Vienna, Austria, ISBN 3-900051-07-0, URL http://www.R-project.org).

### Statistical analyses, profile modeling and chemical diversity

To directly compare behavioral observations (foraging networks at trees and nests) and chemical analyses (chemical networks), we used the quantitative specialization indices *d_i_*′ and *H*_2_′ [43]. The index *d_i_*′ (species-level specialization) describes the exclusiveness of a species, i.e. its quantitative deviation from the overall distribution of all bees on resin sources or of the overall distribution of compounds on all bees. The related network-level specialization index *H*_2_′ characterizes the overall quantitative partitioning of resin sources or chemical compounds across species. Both measures range between 0 (each species uses the same resin sources or has identical chemistry) and 1 (species uses a different set of resins or have unique compounds, i.e. complementary specialization). These indices take the observed variation in number of observations per species into account, using a null model approach. To assess specialization indices for chemical networks, proportions of compounds were multiplied by 1000 and rounded to obtain integers, as *H*_2_′ can only be calculated for integers. *H*_2_′ values are virtually unaffected when matrix cell entries are multiplied in many cases (as tested with two chemical networks for multiplication factors between 10 and 10000), but any multiplication factor >1 prevents calculation of meaningful significance levels for comparisons against null models. Significance levels are therefore only reported for foraging networks where cell entries represent independent counts.

Whereas *H*_2_′ describes the overall degree of chemical partitioning, the significance (*P*-value) and variance explained (*R* ^2^) by intergroup differences were analyzed by Adonis. This test was applied to those datasets where all groups (e.g., bee species, bee colonies) were present and had a sufficient number of replicates (*n*≥3 colonies or individuals). In addition to *H*_2_′, we report mean Bray-Curtis distances among all groups (from the distance matrices underlying Adonis) which also describe general dissimilarity across groups (see Table 1). Conclusions were unchanged for this alternative metric. Hence *H*_2_′ is an appropriate tool to describe and compare both foraging and chemical networks.

We modeled a hypothetical degree of specialization (*H*_2_′_M_) of terpenoid compounds on the bees' chemical profiles for the case that bees simply transfer terpenoids from resins collected to their body surface. We assumed that for bee species *b*, *p_bc_* is the proportion of terpene *c* on its profile (for each bee, ∑ *p_bc_* = 1). For a complete admixture of substances, *p_bc_* is predicted by the proportional distribution of this bee across each resin source *r* (*p_br_*) and the proportion of terpene *c* at each resin source *r* (*p_cr_*). These proportions are summed over all *R* resin types to yield the expected *p_bc_* as
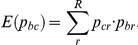



The entire terpene profile of *b* is given as a vector containing a total of *C* terpenes. For the actual as well as hypothetical chemical network, *H*_2_′ was assessed separately for all single terpenoid compounds and for classes of terpenoids (including monoterpenes, sesquiterpenes, sesquiterpenes with functional groups and triterpenes).

We compared the chemical diversity of stingless bees with the chemical diversity of (environmentally derived) fragrances collected by euglossine bees and of the (genetically determined) surface profiles from formicine ants and bumblebees. Data for 15 euglossine bee species were obtained from Thomas Eltz (pers. comm.), who provided an extended dataset including all compounds detected, which is the basis of the study by Zimmermann et al. [50]. For ant species, we used the table compiled by Martin and Drijfhout [51] from which only those 29 species were selected that occur in Central Europe. Bumblebees were collected by Thomas Schmitt and comprised species from Germany and Switzerland. Their chemical profiles were analyzed and characterized by GC-MS using the same methods and criteria as described above for stingless bees. Chemical diversity was simply defined as the total number of different compounds, because concentrations were unavailable for ants and most compounds in euglossines. For a set of species, the cumulative diversity increases with additional species, but the slope saturates depending on the overlap between species. Like in biodiversity studies, we modeled the cumulative diversity curves for all hymenopteran groups using rarefaction of the available data (10000 randomizations) using EcoSim 7 [52].

## Results

### Foraging networks

Stingless bees of different species collected resins from the same tree species. In total, we observed 115 interactions between resin collecting bees and trees. The quantitative resin – bee interaction network showed a very low degree of complementary specialization (*H*_2_′ = 0.20, Fig. 1), suggesting a largely opportunistic collecting behavior of bees. The interaction network did not differ significantly from a random distribution of species (*P* = 0.06). Ten of the 13 bee species collecting resin at trees showed very low degrees of specialization (all *d_i_*′≤0.18). Only *Tetrigona binghami*, *Tetrigona apicalis* and *Geniotrigona thoracica* were slightly more specialized resin foragers (0.31≤*d_i_*′≤0.37). *Tetragonilla collina* was most frequently observed at trees and collected resin from overall twelve different tree species (Fig. 1). Among resin secreting trees, *Shorea xanthophylla* (Dipterocarpaceae) was the most common species (13 tree individuals) and most frequently visited by bees (Fig. 1).

All bee species further collected a similar range of resin colors, again yielding a very low degree of complementary specialization (2007: *H_2_′* = 0.15; 2008: *H_2_′* = 0.27). Within species, colonies did not differ either (2007: *T. collina*: *H_2_′* = 0.09, *Tetragonula melanocephala*: *H_2_′* = 0.18; 2008: *T. collina*: *H_2_′* = 0.23, *T. melanocephala*: *H_2_′* = 0.12, *Tetragonula geissleri/laeviceps* group: *H_2_′* = 0.20).

### Chemical source networks

**Figure 2:**
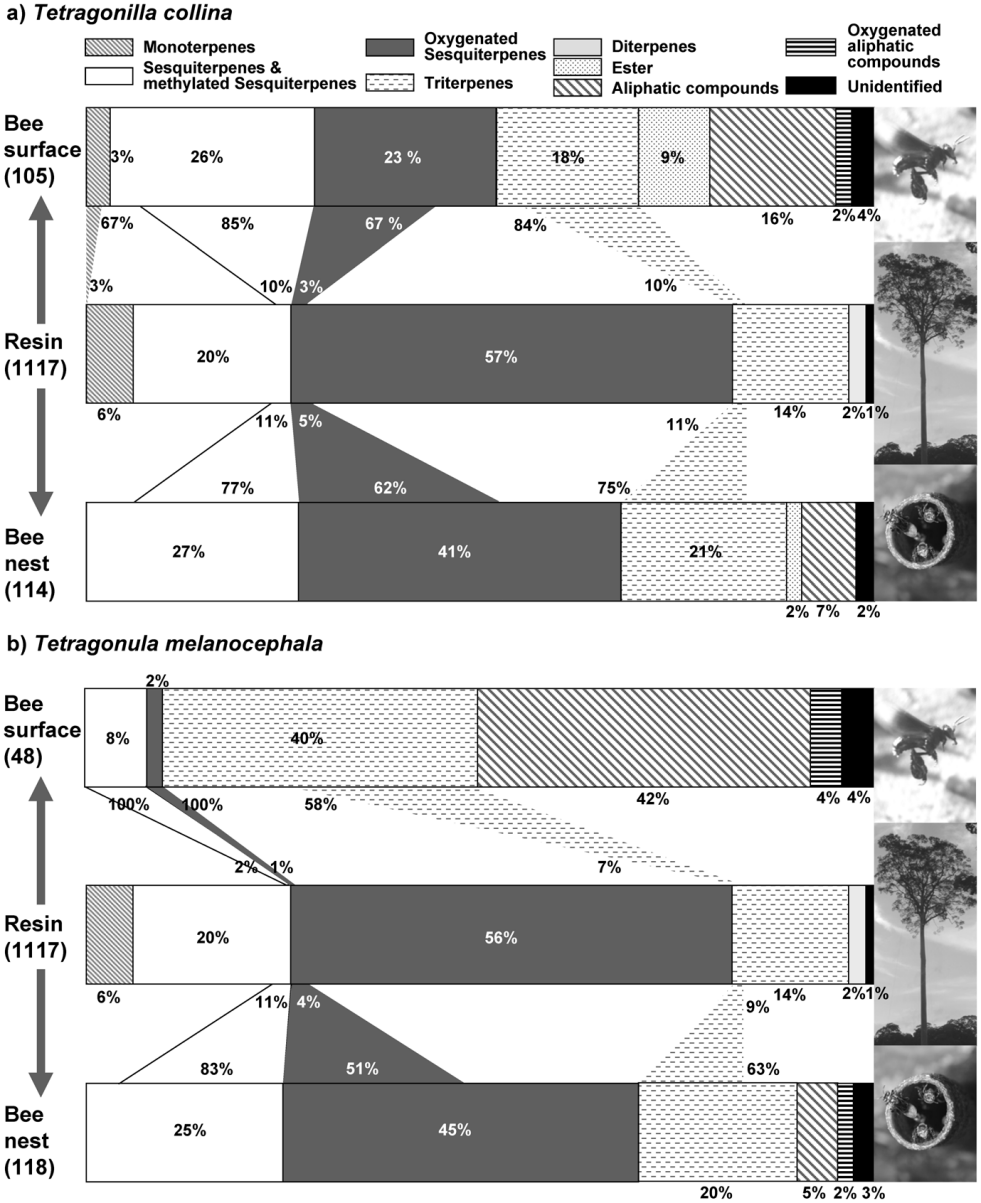
Examples of two bee species for which data was collected from tree resins, nest material and the bees' cuticles: Proportions (based on numbers of compounds) of compound classes in the chemical profile of 14 tree resins (seven tree species, middle), surfaces of individual bees (up) and their nests (below) are shown for (a) *Tetragonilla collina* and (b) *Tetragonula melanocephala*. Proportions of compounds (from particular compound classes) of tree resins that are transferred to bee surfaces/ nests are given above/ below the resin profile. Proportions of compounds (from particular compound classes) that are identical with compounds on bee surfaces/ in nests are given below/ above the profiles of bee surfaces/ nests. Numbers in parentheses give total numbers of compounds on bee surfaces, in bee nests and in resin.

Resin extracts comprised mono-, sesqui-, di- and triterpenes as well as some unknown and very few aliphatic compounds (Fig. 2). Tree species strongly differed both qualitatively and quantitatively in their resin chemistry. Differences were slightly more pronounced for all compounds (*H_2_′* = 0.59) than when compounds were grouped in mono-, sesqui- and triterpenes (*H_2_′* = 0.48). Sesqui- and triterpenes represented the most prominent classes of terpenes (Fig. 1, Fig. 2) and were highly characteristic of dipterocarp trees [14] – the dominant tree family of Southeast Asian forests [53].

### Acquired chemical networks

Most of the terpenoid compounds in the bees' nests and on their body surfaces were identical with compounds found in resin of one or several trees from the small subset of tree species analyzed (60–100% congruence, depending on the species and class of terpenoids), indicating that bees obtain their cuticular terpenes from resin. However, only a small subset of the 1117 terpenes from resins was found in nest material (113 compounds, 0.4–3.7%) and body surface profiles (83 compounds, 0.4–3.0%) of all bee species studied. Overall, the terpene profiles of bee surfaces and nests were dominated by the most prominent resin terpenes: mean proportional concentrations of terpenes were significantly correlated between all tree resin samples and surface profiles of all bee species (Spearman rank correlation: *r*_S_ = 0.31, *P*<0.0001, N = 1117 terpenes), as well as between resin and nests (*r*_S_ = 0.32, *P*<0.0001, N = 1117 terpenes). However, this correlation was less pronounced for the surface profiles of single bee species (*T. collina*: *r*_S_ = 0.26, *P*<0.0001, n = 1117 terpenes; *T. melanocephala*: *r*_S_ = 0.14, *P*<0.0001, n = 1117 terpenes). Moreover, bee species strongly differed in the proportion of terpenoid groups derived from resin and included in their chemical profiles (*H_2_′* = 0.45; shown in detail for *T. collina* and *T. melanocephala*, Fig. 2). Some species (*T. melanocephala*, *Lepidotrigona terminata*, *Pariotrigona pendleburyi* and *Tetragonula fuscobalteata*) even completely lacked sesquiterpenes, whereas all species had triterpenes (see also [20]).

Terpenes in the chemical profiles of nests and bee surfaces were largely identical, but did not completely overlap. Twelve resin-derived terpenes were found on the bees' surfaces but not in their nest material. The bees' nest material had a species-specific chemical composition (*H_2_′* = 0.42). It differed less with regard to resin-derived compounds (terpenes: *H_2_′* = 0.38) than with regard to wax-derived compounds (*H_2_′* = 0.45). The same was true for the bees' body surface profiles when all compounds were taken into consideration (entire profiles: *H_2_′* = 0.50; only non-terpenoids: *H_2_′* = 0.59; only terpenes: *H_2_′* = 0.29).

Given the generalized resin collecting behavior of stingless bees (*H_2_′* = 0.20), the species-specificity of cuticular terpenes (*H_2_′* = 0.26) was higher than would be expected for a simple transfer (contamination) of resin-derived terpenes to bee surfaces (*H_2_′_M_* = 0.17). Moreover, for groups of terpenes, it was substantially higher (*H_2_′* = 0.45) than would be expected (*H_2_′_M_* = 0.14). Sesquiterpenes were, for instance, much reduced in the chemical profile of *T. melanocephala* (8%) compared to their collected resins (from which the mixing model would predict a proportion of sesquiterpenes of 58%).

When the two major terpene classes in the bees' chemical profiles (sesquiterpenes and triterpenes) were analyzed separately, bees appeared more similar (sesquiterpenes: *H_2_*′ = 0.17; triterpenes: *H_2_′* = 0.21), suggesting that all bee species largely obtain the same subset of terpenoid compounds within a given group of terpenes. Hence, stingless bees appear to filter among (e.g., exclude all sesquiterpenes), but not within groups of terpenes.

Within each species, different colonies showed only small differences in their chemical profiles (all *H_2_′*≤0.19), independent of whether terpenoid or waxy compounds were considered (Table 2).

**Table 2:** Foraging and chemical networks analyzed (N1 and N2 give sample sizes for both groups in each network, mBC = mean Bray-Curtis distance).

Number	Network	Year	Location	N1	N2	*H_2_′*	*R^2^*	*P*	mBC ± SD
Foraging networks (n1 - n2)									
1	tree species - bee species	2008	RDC	15	13	0.20	-	-	-
2	bee species - resin color	2007	RDC, KSR, DVC	2*	25	0.12	-	-	-
3	bee species - resin color	2008	RDC, KSR, DVC	3**	21	0.27	-	-	-
4	*Tetragonilla collina* colonies - resin color	2007	RDC, KSR, DVC	4	23	0.09	-	-	-
5	*Tetragonilla collina* colonies - resin color	2008	RDC, KSR, DVC	6	17	0.23	-	-	-
6	*Tetragonula melanocephala* colonies - resin color	2007	RDC, KSR, DVC	4	17	0.18	-	-	-
7	*Tetragonula melanocephala* colonies - resin color	2008	RDC, KSR, DVC	3	7	0.12	-	-	-
8	*Tetragonula geissleri/laeviceps* colonies - resin color	2008	RDC, KSR, DVC	2	9	0.20	-	-	-
Chemical networks (n1 - n2)									
9	tree species - resin compounds (terpenes)	2007	RDC, DVC	7	263	0.59	-	-	0.71±0.26
10	tree species - resin compound groups (terpenes)	2007	RDC, DVC	7	5	0.48	-	-	0.50±0.36
11	nest material - all compounds	2007	KSR, DVC	6	247	0.42	0.90	<0.001	0.70±0.16
12	nest material - wax compounds	2007	KSR, DVC	6	91	0.45	0.86	<0.001	0.69±0.18
13	nest material - terpenoid compounds	2007	KSR, DVC	6	156	0.38	0.90	<0.001	0.66±0.17
14	bee species - all compounds	2007	KSR, DVC	6	194	0.50	0.87	<0.001	0.72±0.16
15	bee species - non-terpenoid compounds	2007	KSR, DVC	6	80	0.66	0.87	<0.001	0.82±0.09
16	bee species - terpenoid compounds	2007	KSR, DVC	6	114	0.29	0.80	<0.001	0.65±0.19
17	bee species - terpenoid compound groups	2007	KSR, DVC	6	5	0.45	0.91	<0.001	0.36±0.26
18	bee species - only sesquiterpenes	2007	KSR, DVC	6	67	0.17	0.39	0.002	0.39±0.14
19	bee species - only triterpenes	2007	KSR, DVC	6	40	0.21	0.66	<0.001	0.42±0.17
20	*Tetragonilla collina* - all compounds	2007	KSR, DVC	9	124	0.08	0.39	<0.001	0.27±0.07
21	*Tetragonilla collina* - non-terpenoid compounds	2007	KSR, DVC	9	49	0.05	0.54	<0.001	0.18±0.09
22	*Tetragonilla collina* - terpenoid compounds	2007	KSR, DVC	9	75	0.13	0.35	<0.001	0.36±0.11
23	*Tetragonula fuscobalteata* - all compounds	2007	KSR, DVC	8	127	0.09	-	-	0.26±0.12
24	*Tetragonula fuscobalteata* - non-terpenoid compounds	2007	KSR, DVC	8	57	0.11	-	-	0.34±0.15
25	*Tetragonula fuscobalteata* - terpenoid compounds	2007	KSR, DVC	8	70	0.08	-	-	0.19±0.11
26	*Tetragonula geissleri/laeviceps* - all compounds	2007	KSR, DVC	2	88	0.03	-	-	0.12
27	*Tetragonula geissleri/laeviceps* - non-terpenoid compounds	2007	KSR, DVC	2	41	0.02	-	-	0.11
28	*Tetragonula geissleri/laeviceps* - terpenoid compounds	2007	KSR, DVC	2	47	0.03	-	-	0.13
29	*Tetragonula melanocephala* - all compounds	2007	KSR, DVC	5	102	0.09	0.60	<0.001	0.25±0.05
30	*Tetragonula melanocephala* - non-terpenoid compounds	2007	KSR, DVC	5	46	0.14	0.58	<0.001	0.35±0.09
31	*Tetragonula melanocephala* - terpenoid compounds	2007	KSR, DVC	5	56	0.06	0.80	<0.001	0.18±0.05
32	*Lepidotrigona terminata* - all compounds	2007	KSR, DVC	4	62	0.17	-	-	0.33±0.09
33	*Lepidotrigona terminata* - non-terpenoid compounds	2007	KSR, DVC	4	38	0.11	-	-	0.20±0.08
34	*Lepidotrigona terminata* - terpenoid compounds	2007	KSR, DVC	4	24	0.19	-	-	0.39±0.15

*T. collina & *T. melanocephala*.

**T. collina, *T. melanocephala* & *T. geissleri/laeviceps*.

Polar compounds in resin samples from corbiculae of *T. collina* did not chemically differ from resin samples directly obtained from the collecting tree (Adonis: *R^2^* = 0.15, *P* = 0.28). The composition of *Agathis borneensis* resin did not change with time neither for directly obtained tree resin (Adonis: *R^2^* = 0.12, *P* = 0.69) nor for *T. collina* corbiculae resin (Adonis: *R^2^* = 0.16, *P* = 0.82).

### Chemical diversity in other hymenopterans

**Figure 3:**
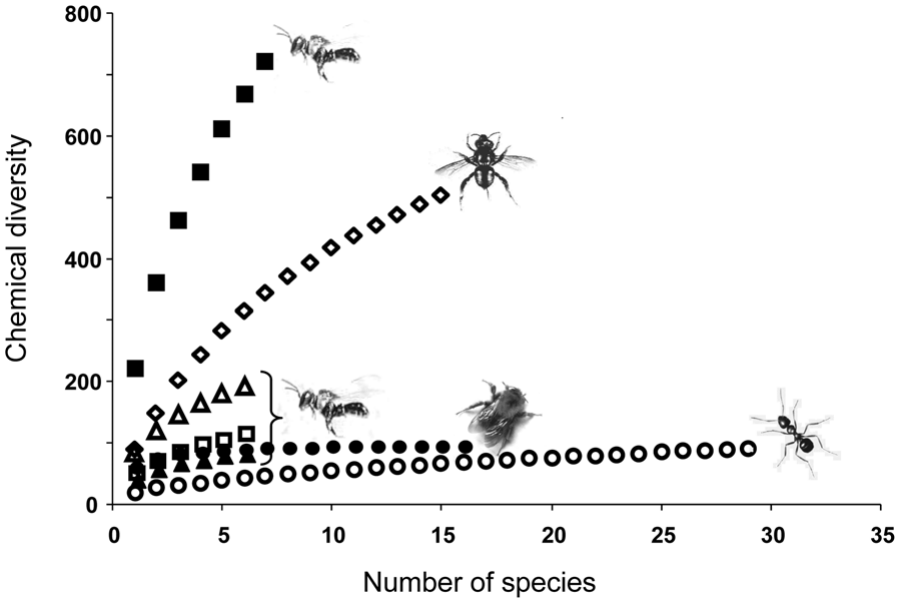
Chemical diversity of the surface profiles from six stingless bee species (squares and triangles), fragrances of 16 euglossine bee species (diamonds) as well as surface profiles of 29 Central European ant species (open circles) and 16 bumblebee species (solid circles). Note that there are two diversity curves for surface profiles of stingless bees, one for all compounds (solid squares) and one for compounds that account for more than 0.05% of the total peak area (open triangles); the reduced compound group is further divided in one curve with only terpenoid compounds (open squares) and one with only non-terpenoid compounds (solid triangles). The bumblebee curve is also based on a reduced dataset using the same threshold and therefore directly comparable to the lower curves of stingless bees, whereas data for ants and euglossine bees have been obtained from other sources (T. Eltz, pers. comm., Martin and Drijfhout 2009).

If all compounds (including substances that accounted for less than 0.05% of the total peak area) were included in the cumulative diversity analysis, stingless bees showed the highest diversity of chemical compounds on their body surface (Fig. 3). Moreover, the diversity curve was far from saturation, indicating that the chemical diversity would strongly increase if additional species were included (Fig. 3). By contrast, surface compounds of ants and bumblebees had a relatively low chemical diversity and a lower slope (Fig. 3). Fragrances of euglossine bees showed an intermediate chemical diversity (Fig. 3).

## Discussion

The chemical diversity of insects comprises both genetically determined and environmentally – particularly plant – derived compounds with the latter fraction depending on the chemical heterogeneity of environmental sources as well as on how they are collected and selected by the insect. Stingless bees are generalized resin collectors with species-specific compositions of terpenes derived from plant resins in their cuticular profiles [20] and their nest profiles. Most of these terpenes could be directly attributed to resin from trees in their habitat (particularly to resins from the dominant dipterocarp trees) even in the small set of tree species studied, but comprised only a subset of the vast amount of terpenes generally found in the tree resins sampled. Along with their genetically determined non-terpenoid compounds, these cuticular terpenes account for a remarkably high chemical diversity in stingless bees. By contrast, ants and bumblebees – which do not or only rarely include environmentally derived compounds in their chemical profiles [54] – show a relatively low chemical diversity. The chemical diversity of fragrances from different orchid bee species (Euglossini) is also relatively high because these fragrances comprise a large variety of predominantly plant derived compounds, but lack genetically determined compounds. In contrast to stingless bees, male orchid bees show a highly specialized collection behavior when collecting fragrances for their courtship bouquets [55]. Here, specialized foraging directly translates into highly species-specific odor bouquets (*H_2_′* = 0.66; data obtained from [50]), rendering any selective reduction or modification of compounds unnecessary. In contrast to euglossine bees, the cuticular terpenes of stingless bees as well as the slope of their diversity curve cannot be explained by direct or passive compound-transfer from resin to bee surfaces. The restricted number of cuticular terpenes on the bees' body surfaces rather suggests that bees are able to ‘filter’ and thus limit the number of resin-derived compounds. Moreover, cuticular terpenes of all bee species are obtained from the same small subset of prominent resin-derived terpenes, but can strongly differ in their quantitative and qualitative composition between different bee species, in particular with regard to terpene groups (*H_2_′* = 0.45). For instance, sesquiterpenes were present in *Tetragonilla collina*, but basically absent in *Tetragonula melanocephala*. Moreover, the nest profile of *T. melanocephala* comprised sesquiterpenes, whereas its surface profile did not. It is therefore likely that stingless bee species are able to specifically ‘filter’ resin-derived compounds, with some species excluding whole compound classes, suggesting that the acquisition of terpenes has a genetic base in these bees. In addition to variation in genetically determined hydrocarbons, bee species-specific terpene profiles (due to selective ‘filtering’) may account for a steeper slope of the diversity curve. Its slope is comparable to the diverse fragrances of euglossine bees, but contrasts with the more similar cuticular profiles of other hymenopterans (e.g.; bumblebees: *H*_2_′ = 0.21). Note that the different experimental setups (e.g., different instruments, columns and temperature programs used for the GC-MS analyses for ants and bees) may account for part of the observed differences. However, analytical procedures are unlikely to explain the pronounced variation in chemical diversity found among the four hymenopteran groups, because all bee groups were analyzed using comparable programs and instruments, and only the ant dataset from Martin and Drijfhout [51] comprised different experimental setups.

The ‘filtering’ process appears to take place within the bees' nests. We did not find chemical differences between resin samples collected from bee corbiculae and samples directly collected from the bees' collecting tree, excluding the possibility that bees add specific enzymes during the collecting process. It thus remains to be investigated where and how precisely the bees obtain their cuticular terpene profiles. Bees may consume and subsequently sequester resin-derived terpenes, as shown for the sawfly larva *Neodiprion sertifer* [35]. Alternatively, bees may be covered by resin due to constant contact with their nest environment or even active application of resin. The species-specific differences in genetically determined chemical surface compounds may then account for variable degrees of evaporation of resin-derived compounds. Different groups of terpenes would then be ‘trapped’ on the surfaces of different bee species.

Mixing environmental and genetic compounds not only results in a higher diversity of compounds, it also increases the number of functions mediated by them. Genetically determined hydrocarbons are known to play a role in the bees' recognition system [56], while resin-derived terpenes in both nest material and chemical profiles protect the bees and their nests against bacteria and fungi [57]. In a humid and warm environment – like the wet tropics – defense against microbial pathogens and infections of their brood and food storage is crucial for the survival of eusocial bees [58], [59]. Cuticular terpenes also deter predators such as ants and termites [41]. Therefore, resin-derived terpenes may have primarily functioned as defense against microbes and predators. Due to their species-specific distribution, they could have become involved in intra- and interspecific recognition as has generally been suggested for primarily defensive compounds in arthropods [17].

Overall, resin and resin-derived terpenes play a fundamental and hitherto largely neglected role in the ecology of tropical stingless bees, directly linking the chemical ecology of trees and bees. Resin-derived compounds increase the chemical diversity of stingless bee profiles – which exceeds levels found in other hymenopterans – and simultaneously expand the functional diversity mediated by them.
